# Supplementary material for: RNA Chaperones Hfq and ProQ Play a Key Role in the Virulence of the Plant Pathogenic Bacterium Dickeya dadantii
Source: Front Microbiol. 2021 Jun 24;12:687484. doi: 10.3389/fmicb.2021.687484 (PMC8264596; doi:10.3389/fmicb.2021.687484)
Supplement: Supplementary file 1 [file Presentation_1.pdf]

Supplementary data

Table S1: List of strains used in this study

Strains	Description	Source
D. dadantii		
3937 (A4922)	Wild-type strain isolated from Saintpaulia ionantha	Kotoujansky A, Lemattre M, Boistard P. Utilization of a thermosensitive episome bearing transposon TN10 to isolate Hfr donor strains of Erwinia carotovora subsp. chrysanthemi. J Bacteriol. 1982;150: 122–131.
Δ*hfq* (A5292)	A4922 hfq::uidA-Kan^R	C. Blanco
Δ*proQ* (A6175)	A4922 proQ::Cm^R	This work
A6170	A4922 + pBBr1-mcs4	This work
A6171	A4922 + pBBr1-mcs4::hfq	This work
A6172	A4922 + pBBr1-mcs4::proQ	This work
A6173	A5292 + pBBr1-mcs4	This work
A6174	A5292 + pBBr1-mcs4::hfq	This work
A6176	A6175 + pBBr1-mcs4	This work
A6177	A6175 + pBBr1-mcs4::proQ	This work
A6178	A5292 + pBBr1-mcs4::proQ	This work
A6179	A6175 + pBBr1-mcs4::hfq	This work
E. coli		
DH5α	F′ϕ80 dLacZΔ(lacZYA-argF)U169 deoR recA1 endA1 hsdR17 (rk-1, mk+) phoA supE44 λ-thi-1 gyrA96 relA1/F′ proAB+lacIqZλM15 Tn10-Tc	Lab collection
Phages		
PhiEC2	General transducing phage of Dickeya dadantii	Resibois et al, 1984

Table S2: Plasmids used in this study

Plasmids	Description	Source
pKD3	Cm^R	Lab collection
pGEM-T	Cloning vector, Amp^R	Promega
pGEM-T-Δ*proQ*- BglII	pGEM-T with the proQ coding region containing BglII site	
pGEM-T-proQ::Cm	pGEM-T with the proQ coding region containing chloramphenicol resistance cassette	This work
pBBR1-mcs4	Amp^R	(Kovach et al., 1995)
pBBR1-mcs4::proQ	pBBR1-mcs4 containing proQ ±500bp	This work
pBBR1-mcs4::hfq	pBBR1-mcs4 containing hfq ±500bp	This work

Table S3: Primers used for genetic constructions.

Primer	Sequence (5'-3')	Description
P1	GTAGCGCGTTACTGTTTGAGCG	Forward primer located 500bp upstream *proQ*
P2	GCTCATCCACGTTTTGCGGCCC	Reverse primer located 500 downstream *proQ*
P3	GGAGATCTGAAATTTCCTGATTACAACGG G	Diverging with end of *proQ* ; contains BglII site
P4+P3'	CCCGTTGTAATCAGGAAATTTCAGATCTA CGGAGGCCAACCTGGGCATGAAC	Diverging with start of *proQ* + reverse complement of P3; contains BglII site
P5	GCTAGCGTAGCGCGTTACTGTTTGAGCG	Forward primer located 500bp upstream proQ; contains NheI site
P6	AAGCTTGCTCATCCACGTTTTGCGGCCC	Reverse primer located 500 downstream *proQ* ; contains HindIII site
P7	GCTAGCGTGTTCATCAGTTTGCGATTGC	Forward primer located 500bp upstream hfq; contains NheI site
P8	AAGCTTCACCAGACGCGTCGCCAGATGG	Forward primer located 500bp downstream *hfq* ; contains HindIII site

Table S4: List of primers used in qPCR

Gene names	Forward primers	Reverse primers
bcsA	CCCGATGGACAGTGAAAAAC	GGCGATAAACAACCCAATGC
celZ	TGCCGCTCTCTTATTTGGAT	CCCCAGCCATTATTACTCCA
fliC	CCCAGACCAACCTGAACAAA	TACCTTCAGCGGTCTGAACC
hfq	TAATGGCATCAAGCTGCAAG	TCAGCGTCATCACTTTCCTG
hrpN	TACGATTAAAGCGCACATCG	GTATTGAGCGACGCACCAAG
kdgK	AACACCGCCGTCTACATTTC	GGCATCGTTACGCCAGTAGT
outC	CTGCTGATGCTGCTCTTTTG	AGAAACGCCGAATAGCGTAA
pelD	TTGTGGAAGGTAACGCGCAGTTTG	ATGGCAAATTCACCAACGGCTCTC
pelE	AGCGAATTCAAAGCAGCACT	GGCGTTTCGATGTACAGGTT
proQ	TCTCCGTCATCCGAAAAATC	GGAAGCCAGTTGTACCCTGA
prtB	AAAGCGGCAAATCTGACCTA	TTTTGATTGGGGCTGACTTC
prtC	ATGACGCTCAACACGCATTA	AGCTGACCGACTGCAGAAAT
rhlA	GCATATTTCCGATCCTGCAC	CCCAGGAAATCGACAGGATA

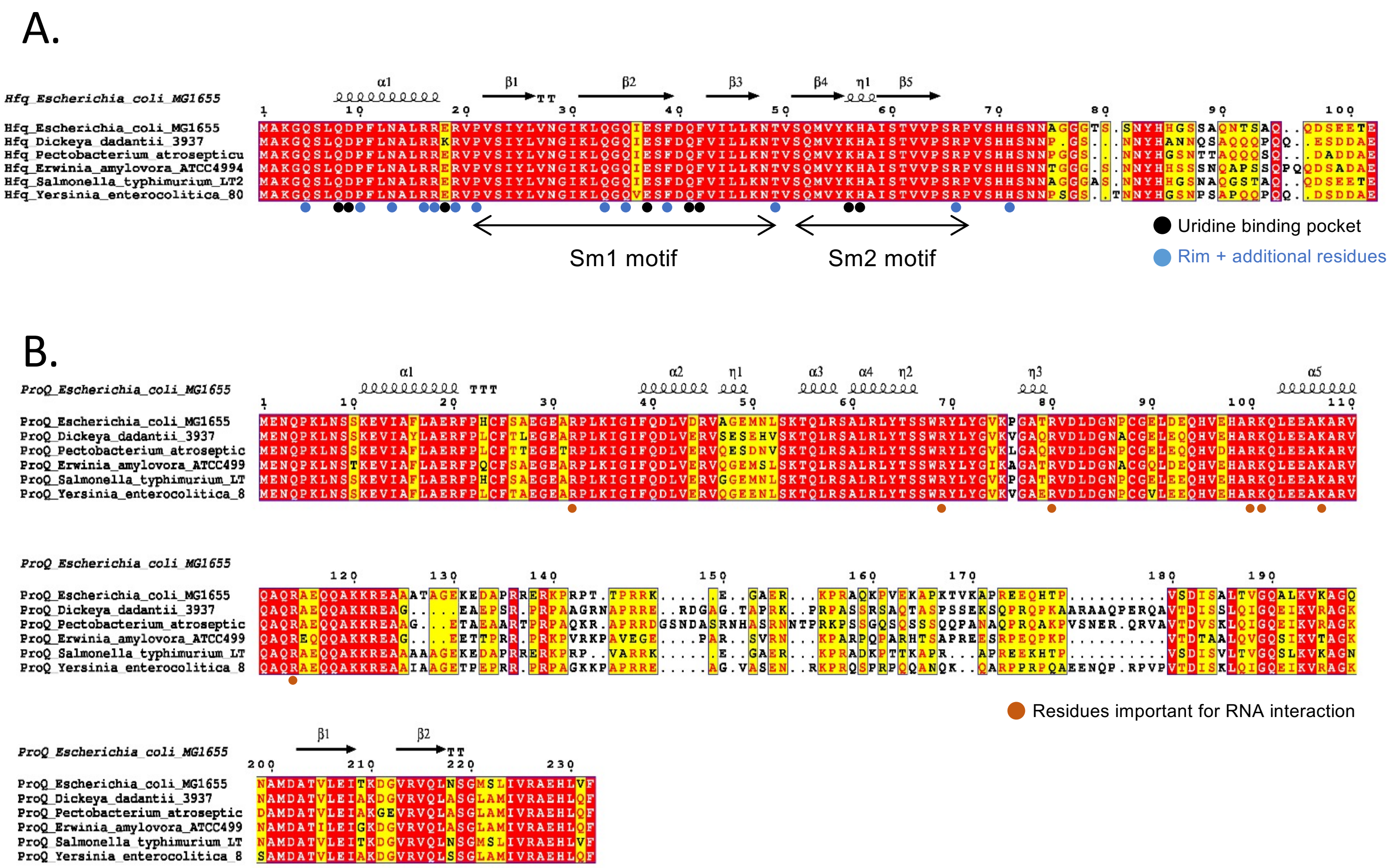

Figure S1: Sequence alignment and secondary structure prediction of Hfq (A) and ProQ (B) protein homologs identified in *Escherichia coli, Dickeya dadantii, Pectobacterium atrosepticum, Erwinia amylovora, Salmonella typhimurium* and *Yersinia enterocolitica.*

A.

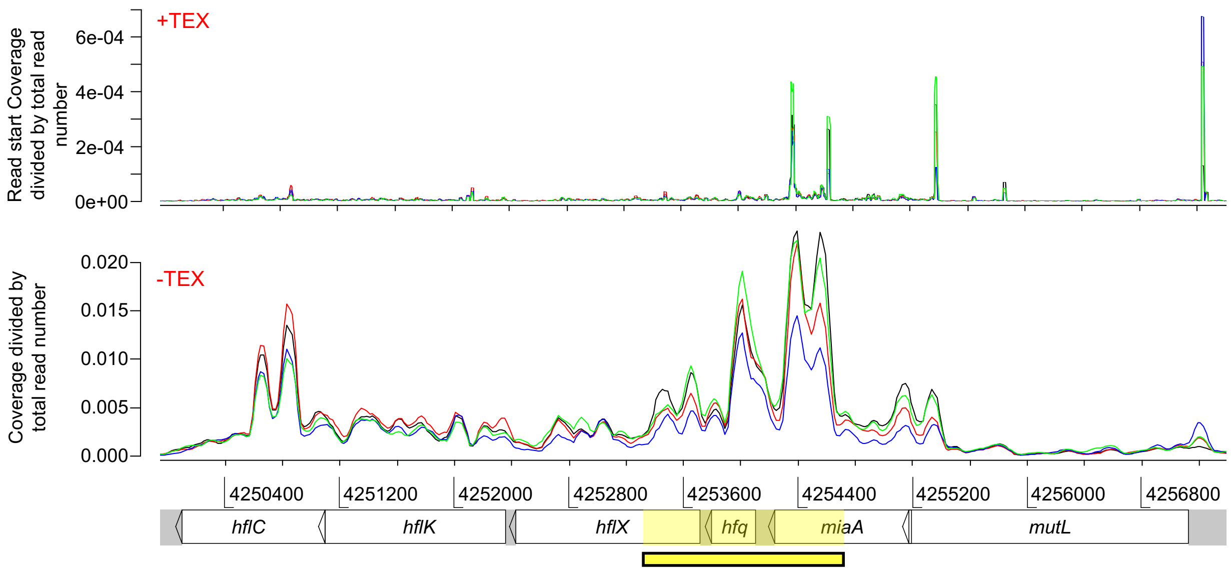

B.

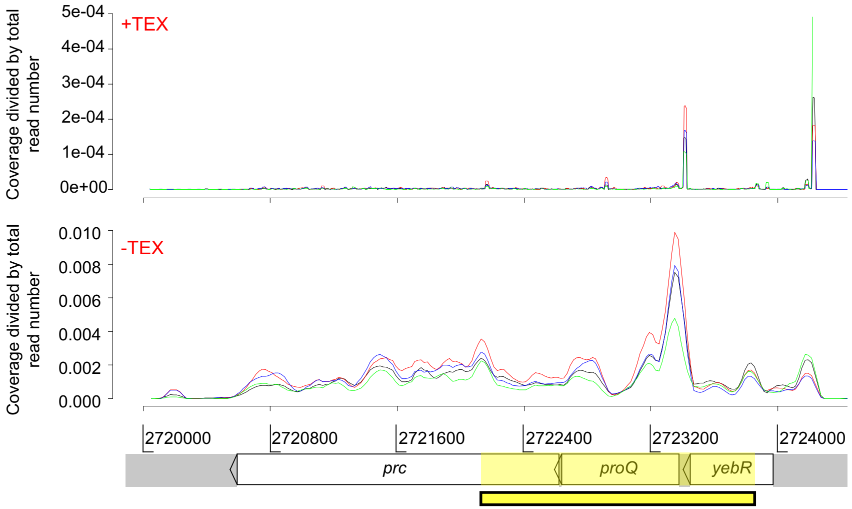

C.

Line color	Growth phase	Stress
Blue	Stationary	No
Red	Exponential	No
Green	Stationary	Yes
Black	Exponential	Yes

Figure S2: Genomic context and expression profiles of the *hfq* (A) and *proQ* (B) genes in *Dickeya dadantii* 3937. Genomic coordinates are given in the x-axis at the bottom of the figures. The normalized intensities (read coverage for the -TEX library and read start coverage for the +TEX library) are represented in the y-axis. Highlighted regions correspond to fragments used for plasmid complementation. Line colors represent the expression profiles, with sequencing conditions detailed in C.

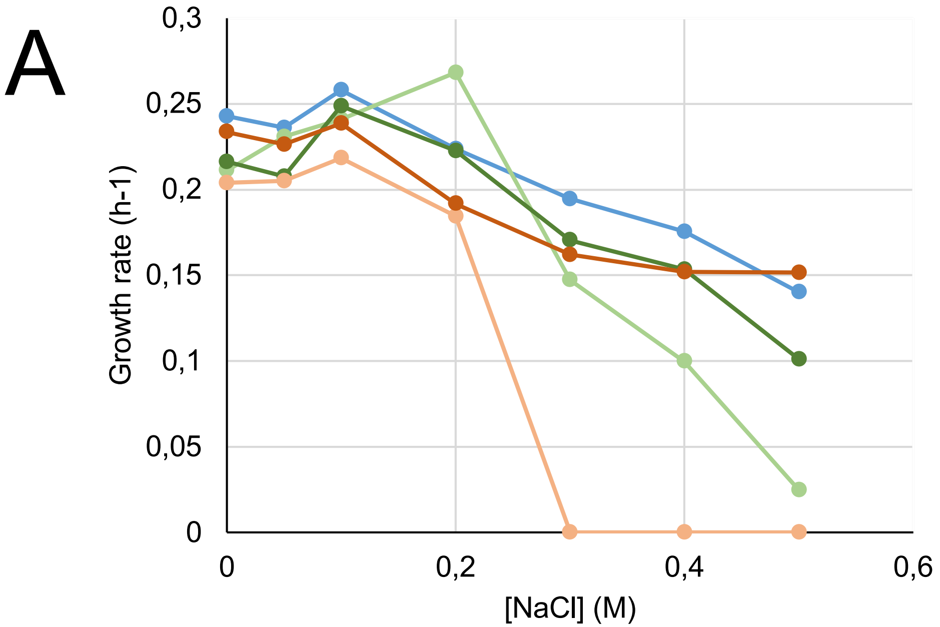

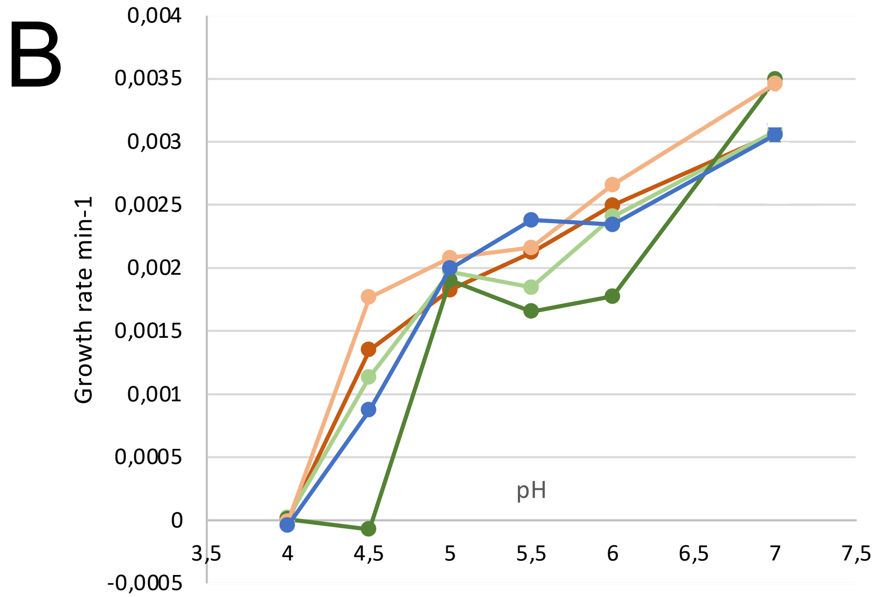

Strain	Δ*hfq*	Δ*hfq*	WT	Δ*proQ*	Δ*proQ*
Plasmid	Control	*hfq*	Control	Control	*proQ*

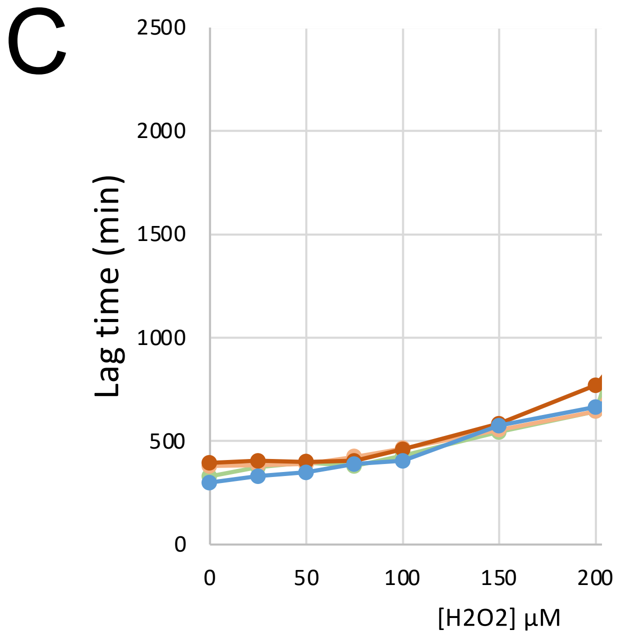

Figure S3: Growth of the wild type, mutant and complemented strains in M63 minimal medium with stress. Overnight bacterial precultures in M63 with sucrose as the carbon source and ampicillin (to maintained plasmid into the cells) were diluted to an OD_{600} of 0.03 in a similar medium with $CaCl_2$ 0.1 mM + polygalacturonic acid (PGA) 0.025 % w/v. A. Osmotic stress was induced by adding different concentrations of NaCl in the medium. OD_{600} measurements of the culture were made at regular intervals to determine growth rates. . A. Osmotic stress was induced by adding different concentrations of NaCl in the medium. OD_{600} measurements of the culture were made at regular intervals to determine growth rates. B The pH effect on growth rate was analysed using M63 with sucrose buffered with malic acid at different pH ranging from 4.0 to 7.0 (abscissa). C Resistance to oxidative stress was analysed in the same medium by adding H_2O_2 concentrations ranging from 25 to 200 µM (abscissa). The lag time is represented instead of the growth rate because after the degradation of H_2O_2 by bacterial catalases, the growth rates are similar.

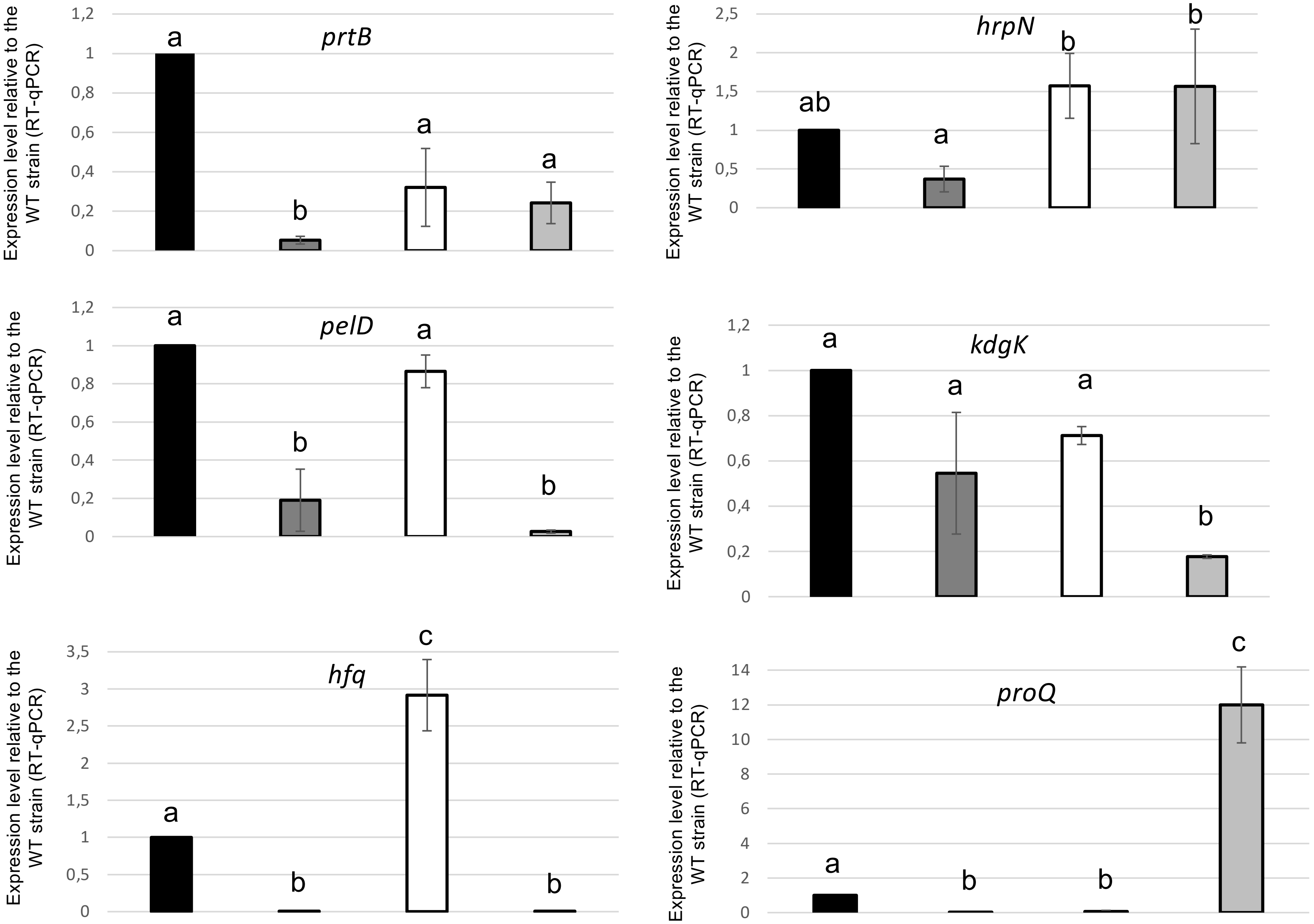

Strain	WT	Δ*hfq*Δ*proQ*	Δ*hfq*Δ*proQ*	Δ*hfq*Δ*proQ*
Plasmid	Control	Control	*hfq*	*proQ*

Strain	WT	Δ*hfq*Δ*proQ*	Δ*hfq*Δ*proQ*	Δ*hfq*Δ*proQ*
Plasmid	Control	Control	*hfq*	*proQ*

Figure S4: Expression levels in the double mutant strain and the double mutant strain complemented by Hfq or ProQ.

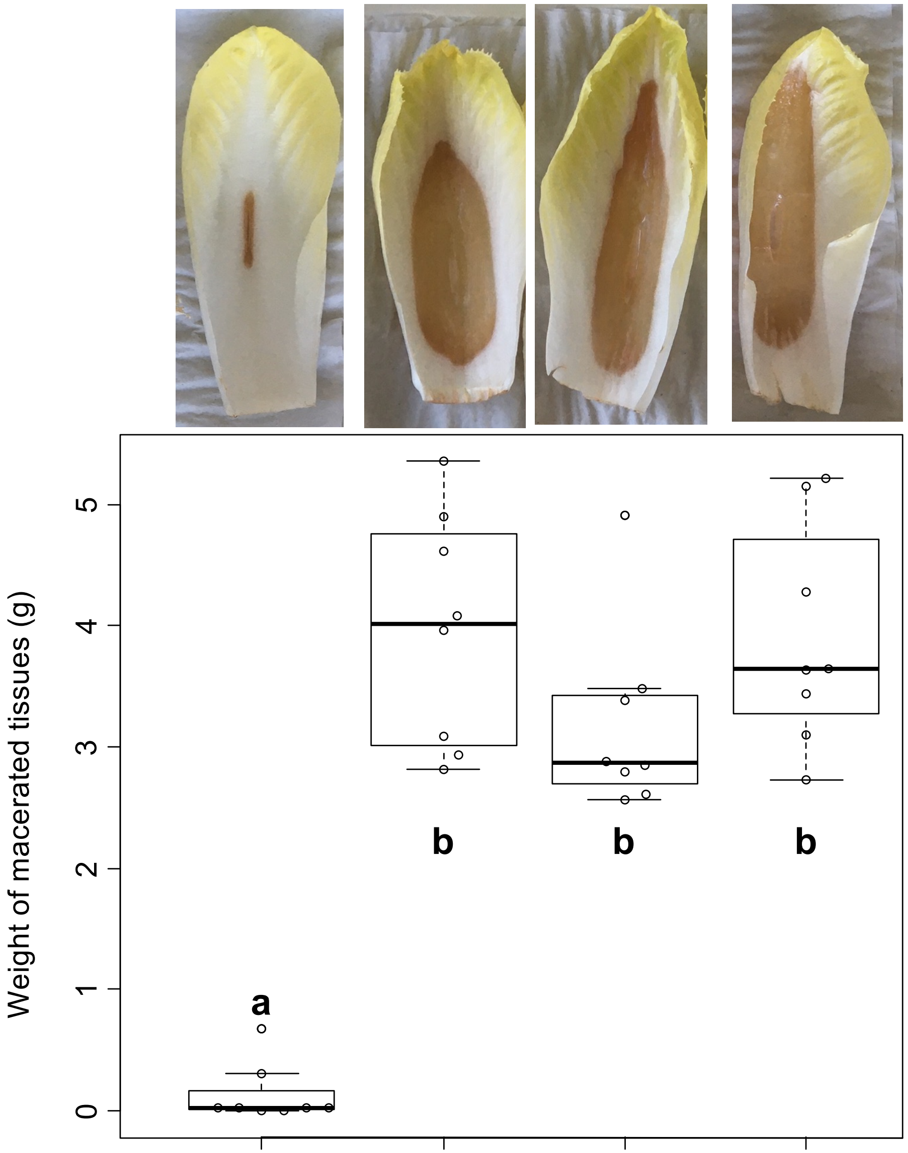

Strain	Δ*hfq*	Δ*hfq*	Δ*proQ*	Δ*proQ*
Plasmid	Control	*proQ*	Control	*hfq*

Figure S5: *D. dadantii* virulence assays 48h post infection. Virulence was evaluated on the *proQ* mutant with heterologous expression of *hfq,* and on the *hfq* mutant with heterologous expression of *proQ*. Chicory leaf assays were performed as described in the Materials and methods section with an incubation time of 48h, and weights of macerated tissues were measured. Representative examples of symptoms induced were shown
